# Human Adipose Derived Stem Cells Induced Cell Apoptosis and S Phase Arrest in Bladder Tumor

**DOI:** 10.1155/2015/619290

**Published:** 2015-01-27

**Authors:** Xi Yu, Boxing Su, Peng Ge, Zicheng Wang, Sen Li, Bingwei Huang, Yanqing Gong, Jian Lin

**Affiliations:** ^1^National Urological Cancer Center, Beijing 100034, China; ^2^Department of Urology, Peking University First Hospital and the Institute of Urology, Peking University, Beijing 100034, China; ^3^Beijing Shunyi District Hospital, Beijing 101300, China; ^4^Qinzhou First People's Hospital, Qinzhou 535000, China

## Abstract

The aim of this study was to determine the effect of human adipose derived stem cells (ADSCs) on the viability and apoptosis of human bladder cancer cells. EJ and T24 cells were cocultured with ADSCs or cultured with conditioned medium of ADSCs (ADSC-CM), respectively. The cell counting and colony formation assay showed ADSCs inhibited the proliferation of EJ and T24 cells. Cell viability assessment revealed that the secretions of ADSCs, in the form of conditioned medium, were able to decrease cancer cell viability. Wound-healing assay suggested ADSC-CM suppressed migration of T24 and EJ cells. Moreover, the results of the flow cytometry indicated that ADSC-CM was capable of inducing apoptosis of T24 cells and inducing S phase cell cycle arrest. Western blot revealed ADSC-CM increased the expression of cleaved caspase-3 and cleaved PARP, indicating that ADSC-CM induced apoptosis in a caspase-dependent way. PTEN/PI3K/Akt pathway and Bcl-2 family proteins were involved in the mechanism of this reaction. Our study indicated that ADSCs may provide a promising and practicable manner for bladder tumor therapy.

## 1. Introduction

Bladder tumor (BT) is the 11th most common cancer and the 14th leading cause of cancer deaths worldwide. It is estimated that about 386,300 people are diagnosed with BT and 150,200 died of this disease in 2012 [1]. The morbidity of male is more than 3 times higher than female and this disparity is more obvious in developed countries. So far, well established risk factors for BT include tobacco use, infections with Schistosoma haematobium, and occupational exposure to aromatic amines, chronic irritation, and polycyclic aromatic hydrocarbons [2, 3]. Most non-muscle-invasive BT is generally treated by transurethral resection of bladder tumor (TUR-BT), followed by adjuvant intravesical therapy. Muscle-invasive cases prefer radical cystectomy and lymphadenectomy followed by adjuvant chemotherapy or radiotherapy. The treatment scheme of BT is expensive and brought huge economic burden to patients. Are there other means that would improve prognosis?

Mesenchymal stem cells (MSCs) have received much attention in recent years owing to their capacity to differentiate into many other cell types including bone, cartilage, stroma, adipose, muscle, tendon, and connective tissue [4, 5]. Recently, more and more studies have found out that MSCs can secrete various cytokines and chemokines which affect the proliferation of tumor cells. MSCs have been proved to increase the growth of colon (KM12SM) [6], prostate [7], lung, or glioma (H460 or U87MG) [8] cancer cells, while inhibiting the growth of pancreatic cancer cells [9], Kaposi sarcoma [10], hepatoma (H22), and non-Hodgkin's lymphoma (SKW6.4 and BJAB) [11]. But it is difficult to obtain MSCs. Adipose tissue, containing a stroma which is easily isolated, is derived from the embryonic mesenchyme. Many studies have identified a putative stem cell within the adipose stromal compartment termed ADSCs which share the same characteristic with MSCs and can differentiate toward the adipogenic, osteogenic, chondrogenic, and myogenic lineages [12].

There are debates about the ability of ADSCs to support or suppress tumor cell proliferation [8, 13–15]. Unfortunately, there are few reports about BT. The purpose of this investigation was to investigate the effects of ADSCs on the growth of bladder cancer cells and to explore the underlying mechanisms. In this paper, we used direct and indirect coculture to detect whether ADSCs may stimulate or inhibit cancer cell growth. If the ADSCs exert an inhibitory effect on cancer cells, it may potentially be used to treat currently incurable BT patients.

## 2. Materials and Methods

### 2.1. Ethics Statement

Human adipose tissues were obtained from subcutaneous fat of patients who underwent radical nephrectomy at Department of Urology, Peking University First Hospital. All patients signed informed consent. This study was approved by Human Research Ethics Committee of Peking University First Hospital (approval ID: 2014[835]).

### 2.2. Chemicals and Reagents

Collagenase I, dexamethasone, ascorbate-2-phosphate, pantothenic acid, and insulin-transferrin-sodium selenite supplement were purchased from Sigma Aldrich (St. Quentin Fallavier, France). Trypsin, Dulbecco's modified Eagle's medium (DMEM), penicillin, streptomycin, and phosphate buffered saline (PBS) were provided by Hyclone (Cergy-Pontoise, France). Fetal bovine serum (FBS) for ADSCs was purchased from Gibico (Paris, France). LY294002 was purchased from Cell Signaling Technology (CST, Beverly, MA, USA).

### 2.3. ADSCs Preparation and Culture

The adipose tissues were washed carefully with sterile PBS to remove debris and red blood cells. Then we cut them into tiny pieces with scissors. The pieces were treated with 0.1% collagenase I in DMEM at 37°C for 60 min with gentle agitation. Then the mixture was centrifuged for 10 min at 1000 r/min. The cellular precipitate was resuspended in DMEM with 10% FBS and then filtered through a 100 m mesh filter to remove debris. The filtrate was plated onto cell culture plates and maintained in an incubator at 37°C with 5% CO_2_. The medium was changed every two days. Cells at passages 3~6 were used for experiments.

### 2.4. Adipogenic Differentiation and Oil Red Staining

Cells were seeded in expansion medium at a density of 2 × 10^4^/cm^2^. When reaching 90% confluence, adipogenesis was induced by adipogenic medium (AM): DMEM supplemented with 10% FBS, 0.5 mM isobutyl-methylxanthine (IBMX), 1 mM dexamethasone, 10 mM insulin, 200 mM indomethacin, and 1% antibiotic/antimycotic for 2 weeks [12, 16]. Prior to staining, the cells were fixed in 4% formaldehyde for 60 min at room temperature and washed with 70% ethanol. The cells were incubated in 2% (wt/vol) Oil Red O reagent for 5 min at room temperature. Excess stain was removed by washing with 70% ethanol followed by several changes of distilled water. The cells were then counterstained for 2 min with hematoxylin.

### 2.5. Osteogenic Differentiation and Von Kossa Staining

Cells were seeded in expansion medium at a density of 2 × 10^4^/cm^2^. When reaching 90% confluence, they were induced in the following osteogenic medium (OM) for 2 weeks: DMEM supplemented with 10% FBS, 0.1 mM dexamethasone, 50 mM ascorbate-2-phosphate, 10 mM b-glycerophosphate, and 1% antibiotic/antimycotic [12, 16]. For Von Kossa staining, cells were incubated in OM for 4 weeks and then fixed with 4% paraformaldehyde for 60 min at room temperature. The cells were rinsed with distilled water and then overlaid with a 1% (wt/vol) silver nitrate solution for 30 min in the absence of light. The cells were washed with distilled water several times and developed under UV light for 60 min. In the end, the cells were counterstained with 0.1% eosin in ethanol.

### 2.6. Cell Lines

Bladder cancer cell lines studied included EJ and T24. These cell lines were cultured at 37°C in 5% CO_2_ and routinely maintained in DMEM supplemented with 10% FBS, 1% penicillin G, and 1% streptomycin.

### 2.7. Cell Number Assessment

Before serum starvation for 16 hours, EJ and T24 cells were cultured in complete DMEM in 6-well plates for 24 h. Experimental cells were cocultured with ADSCs in a transwell membrane with 1.0 *μ*m holes for another 48 hours. Control cells were cultured with cancer cells. Cell growth was measured by cell counting using a cell counter model (Muse).

### 2.8. Cell Viability Assay

EJ and T24 cells were cultured in complete medium in 96-well plates for 24 h, before serum starvation for 16 hours. Experimental cells were cultured in ADSC-CM for 48 hours in triplicate. Control cells were cultured in cancer cells conditioned medium. Cell viability was then measured by MTS assay, according to the manufacturer recommendations (CellTiter96 AQueous Assay, Promega, France). To draw the cell growth curve, cells were cultured for 120 h and measured by MTS assay every 24 h.

### 2.9. Flow Cytometry

To measure apoptosis, an FITC annexin V apoptosis detection kit (BD PharMingen, 556547) was used according to the manufacturer's instructions. The cells were stained with annexin V/PI and detected with flow cytometry. All flow cytometry data were analyzed with FlowJO software.

To measure cell cycle, a DNA content quantitation assay (cell cycle) kit (KeyGEN, KGA512) was used according to the manufacturer's instructions. The cells were stained with PI and detected with flow cytometry. All flow cytometry data were analyzed with ModFit software.

### 2.10. Colony Formation Assay

Digest the cells at logarithmic growth phase and dilute the suspension at multiple grades. For each cell group, 400 cells were seeded onto the plate containing 5 mL 37°C prewarmed broth dish and gently rotated to be evenly dispersed and then incubated in 37°C and 5% CO_2_ humidified incubator for 2~3 weeks. When visible cloning appeared in the well, the culture was terminated. Discard the supernatant carefully and dip the dish two times with PBS. Add 5 mL 4% paraformaldehyde to fix cells for 15 minutes. Remove the fixative and add appropriate amount of crystal violet dye for 10 to 30 minutes; then wash away the stain slowly with water and air drying. Clones greater than 10 cells were counted with the microscope (low magnification).

### 2.11. Wound-Healing Assay

Cells were seeded in 6-well plates and cultured at concentrations to yield 90~95%. Cells were then scratched with a 200 *μ*L pipette tip and washed three times with PBS. Medium containing 1% FBS and ADSC-CM were added to the wells. Wound areas were examined after 0 h, 12 h, and 24 h of incubation under an OLYMPUS inverted microscope connected to a DXM1200 digital camera (Nikon, Tokyo, Japan).

### 2.12. Western Blotting

Whole-cell lysates were prepared in lysis buffer (50 mM Tris-HCl, pH 7.4, 150 mM NaCl, 1 mM EDTA, 1% NP-40, and 0.1% sodium dodecyl sulfate), quantified, and loaded onto SDS-PAGE. After electrophoresis, proteins in the gel were transferred to a nitrocellulose membrane and incubated with primary antibodies at 4°C overnight. The membrane was incubated with HRP-conjugated secondary antibodies for 1 h at room temperature and then washed with TBST. The antibody complexes in the immunoblots were detected by chemiluminescence using an HRP substrate (Millipore, Bedford, MA, USA; WBKLS0100) and visualized using a G:BOX Chemi Gel Documentation System (Syngene, Frederick, MD, USA).

### 2.13. Statistical Analysis

All data was analyzed by SPSS software (version 18.0). The results were expressed as the mean±SD. Statistical analyses were performed using Student's *t*-test. The significance level was set at 0.05.

## 3. Results

### 3.1. Characteristics of Adipose Derived Stem Cells

**Figure 1:**
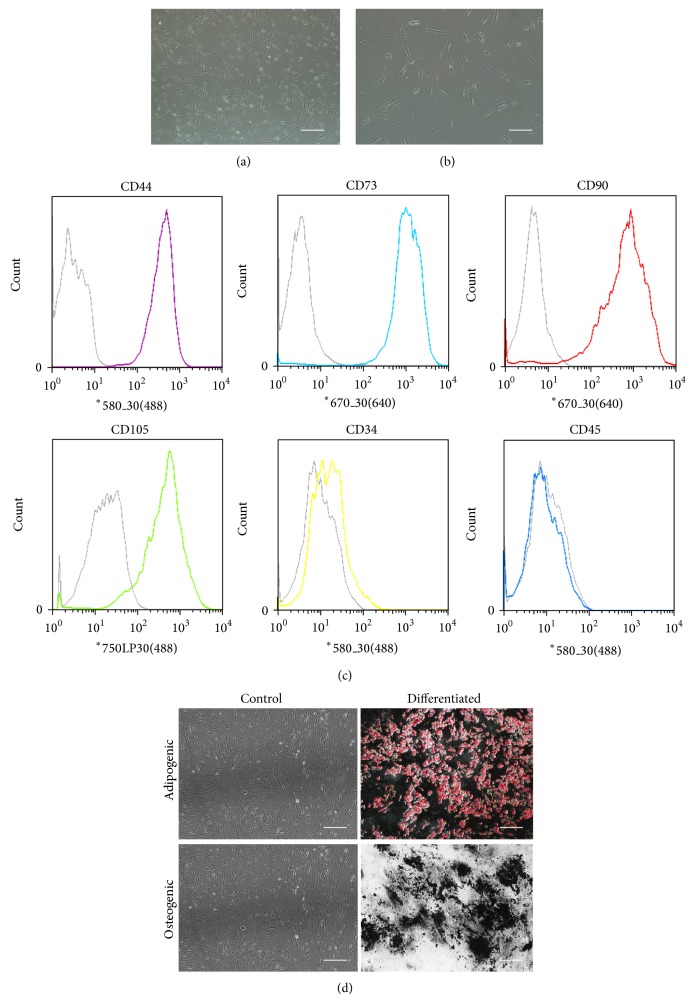
Characterization of ADSCs. ADSCs exhibited a flattened fibroblast-like morphology. Observed under a microscope of 40x (a) and 100x (b). Immunophenotype of ADSCs (c). ADSCs were stained with antibodies against the indicated antigens and analyzed by flow cytometry. Representative histograms are shown as colored lines, and the respective isotype controls are shown as a gray line. ADSCs were positive for CD44, CD73, CD90, and CD105 and negative for CD34 and CD45. ADSCs are able to differentiate into adipogenic and osteogenic lineages, as shown by staining with Oil Red O and Von Kossa, respectively (d). Undifferentiated (control) ADSCs are not marked with the stains. Scale bars, 100 *μ*m ((a), (d)) and 50 *μ*m (b).

After 3 days of growing from the initial plating, the fibroblastoid cells formed a monolayer. Then we replaced the medium every two days. The fibroblastoid cells were termed ADSCs. Cells exhibited homogeneous shapes when it came to the second or third generation (Figures 1(a) and 1(b)). To characterize the ADSCs population, CD marker profile was examined by flow cytometry. As reported [12, 16–18], ADSCs expressed CD44, CD73, CD90, and CD105. In contrast, no expression of CD34 and CD45 was observed in either of the cultures (Figure 1(c)). And ADSCs are able to differentiate into adipogenic, osteogenic lineages (Figure 1(d)).

### 3.2. ADSCs Inhibited EJ and T24 Cells Proliferation and Viability

**Figure 2:**
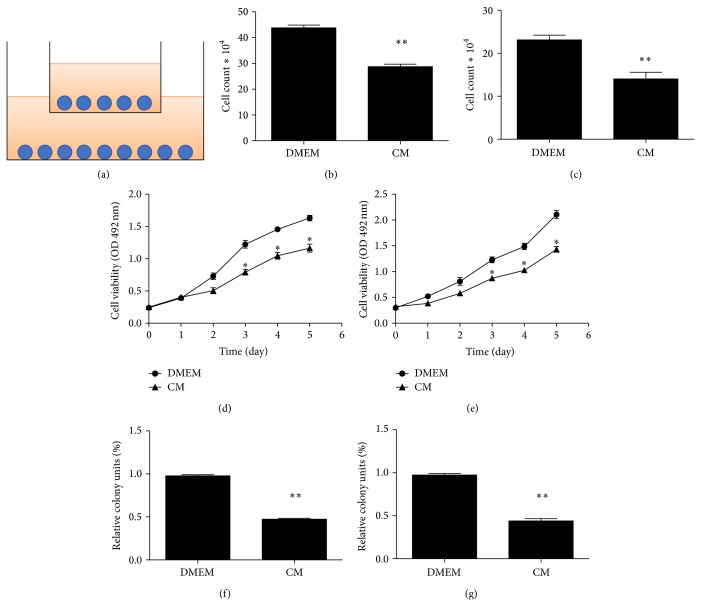
The effect of ADSCs on the growth of human bladder cancer cells. (a) ADSCs and bladder cancer cells were cultured in a transwell membrane with 1.0 *μ*m holes, the upside was ADSCs, and EJ or T24 cells were below. The number of EJ (b) and T24 (c) cell lines was less in the treated group than the control group. Data are shown as the means ± SD (^**^ *P* < 0.01 versus control). Three independent experiments were conducted. The effect of ADSC-CM on the viability of EJ (d) or T24 (e) cells. EJ and T24 cells viability was inhibited. Data shown were the mean ± SD of three independent experiments (^*^ *P* < 0.05 versus control). Treatment of ADSC-CM led to a significant reduction in the number of EJ (f) or T24 (g) colony formation units relative to control cells (^**^ *P* < 0.01 versus control).

To determine effect of ADSCs on EJ cells and T24 cells growth in vitro, we evaluated the viability and progression of cancer cells exposed to ADSCs. The ADSCs were used for coculture with cancer cells after three passages in the presence of 1 : 1 ratio of cancer cells (Figure 2(a)). Cell proliferation rate decreased significantly in the experimental group when compared with the control group after 48 h (Figures 2(b) and 2(c)).

The result from the indirect cell-to-cell contact in our experiment prompted us to infer that maybe some soluble factors were released from ADSCs and could have an effect on cancer cells viability. In fact, soluble factors had been demonstrated to regulate the immunosuppressive effect of human ADSCs previously [19]. As shown in Figures 2(d) and 2(e), ADSC-CM could inhibit the viability of EJ and T24 cells, while as a control conditioned medium from cancer cells alone did not significantly impair cancer cells viability.

We also determined the proliferation of EJ and T24 cells treated with ADSC-CM using colony formation assay. We found a significant reduction of colony formation number in treated group compared with control group (Figures 2(f) and 2(g)).

### 3.3. ADSC-CM Suppressed Migration of EJ and T24 Cells

**Figure 3:**
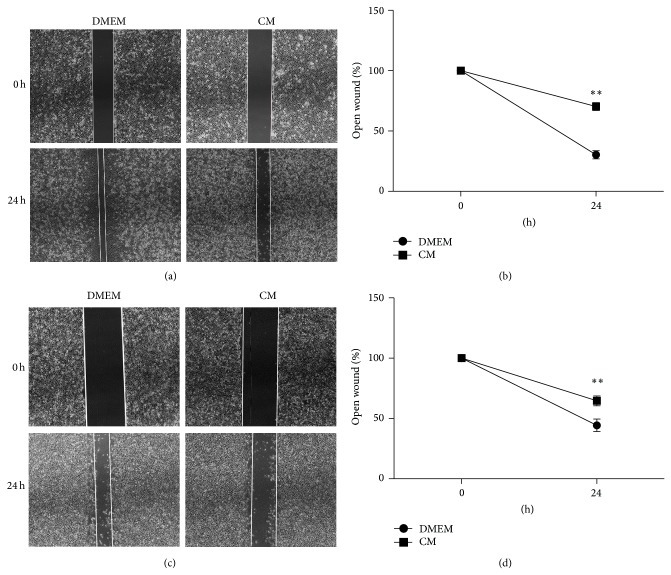
ADSC-CM suppresses migration of EJ and T24 cells. Representative images of wound gaps in ADSC-CM treated EJ (a) or T24 (c) cells and DMEM treated cells at different time points. Original magnification: ×40. Pictures captured at 0 or 24 h represented width of open wound compared with 0 h (^**^ *P* < 0.01).

To evaluate the effect of ADSC-CM on the motility of cancer cells, we performed a wound-healing assay. After incubating for 24 h, we found the migrating cells in the treated groups were significantly reduced compared to control groups (Figures 3(a), 3(b), 3(c), and 3(d)).

### 3.4. Induction of Apoptosis of Cancer Cells by ADSC-CM

**Figure 4:**
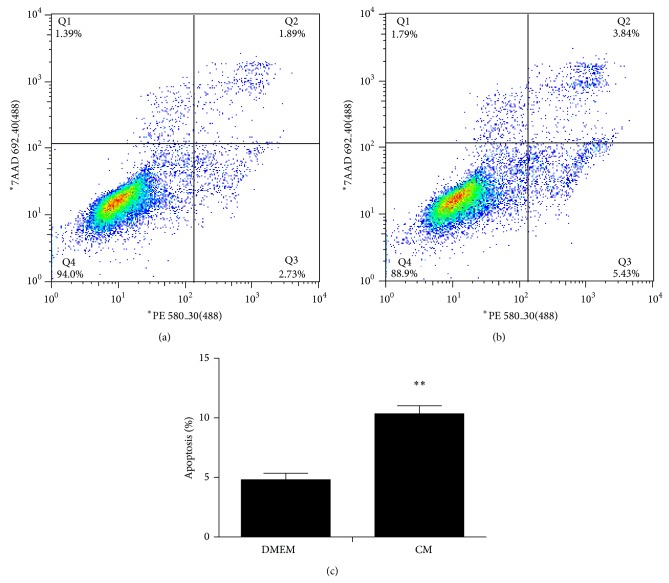
ADSC-CM treatment induces apoptosis in T24 cell line. Cells were double-stained with annexin V and PI and analyzed by flow cytometry. The gate setting distinguished between living (bottom left), necrotic (top left), early apoptotic (bottom right), and late apoptotic (top right) cells. T24 cells were treated with DMEM (a) as a control. Apoptosis was induced by ADSC-CM treatment (b). The apoptosis of T24 cells (c) was increased in the test group more than the control group from three independent experiments (^**^ *P* < 0.01 versus control).

As mentioned above, ADSC-CM inhibited cancer cell growth; then we speculated whether ADSC-CM also induced cell apoptosis. With a treatment of 48 h, we observed increased percentage of early apoptotic cells as well as the late apoptotic cells in treated group (Figures 4(a), 4(b), and 4(c)). Consistent with the MTS assay, the results revealed that ADSC-CM induced cellular apoptosis.

### 3.5. ADSC-CM Induces S Phase Cell Cycle Arrest

**Figure 5:**
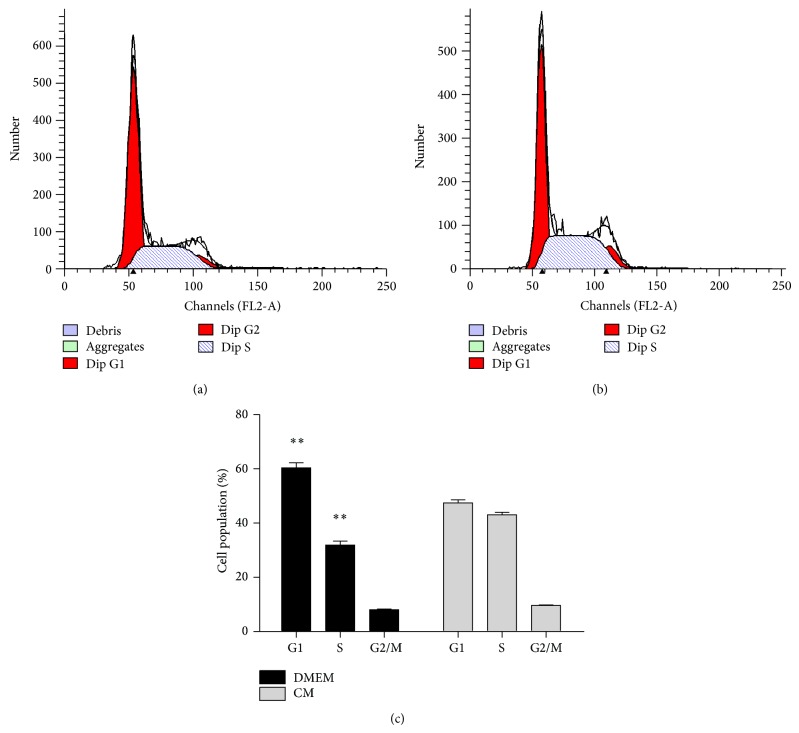
Cell cycle analysis of T24 bladder cancer cells. Cells were treated with DMEM (a) or ADSC-CM (b) for 48 h and then stained with propidium iodide. The DNA content was analyzed by flow cytometry. G1, S, and G2/M indicate cell cycle phase. (c) It shows an obvious S phase arrest with a concomitant decrease of cell number in G1 phase of the cell cycle. The percent of S phase was quantified from three independent experiments (^**^ *P* < 0.01 versus control).

Treatment of T24 cells with ADSC-CM resulted in inhibition of cells growth and induced apoptosis, so we considered the possibility that it may involve an arrest of cells at a specific check point in the cell cycle. In consistence with our hypothesis, compared with the control group, ADSC-CM treatment leaded to an appreciable arrest in S phase of the cell cycle. Cell cycle analysis showed that the S phase population of the control cells was 32.85% (Figure 5(a)) and the percentage of cells in S phase increased to 42.36% (Figure 5(b)) after 48 h treatment. This increase in S phase cell population was accompanied with a decrease of cell number in G1 phase.

ADSC-CM induced apoptosis was mediated by Bcl-2 family modulation and caspase activation.

**Figure 6:**
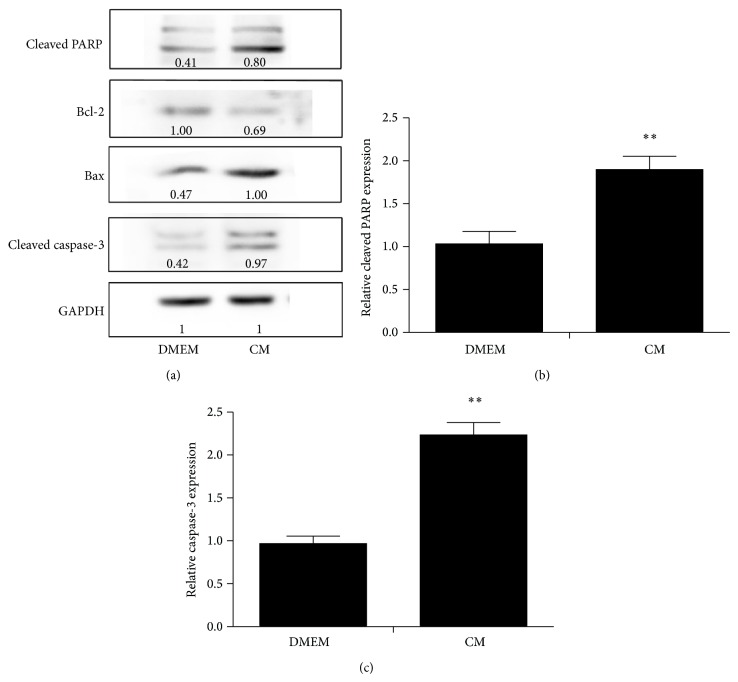
After treatment with ADSC-CM, the expression of antiapoptotic gene products Bcl-2 was inhibited but the expression of apoptotic gene product Bax was upregulated (a). Cleaved PARP (b) and Caspase-3 (c) showed a higher expression in the ADSC-CM treated group. Relative gray values of each protein band were marked under the bands which were measured by Image J software. Data presented are the mean ± SD of three independent experiments (^*^ *P* < 0.05).

To further understand this cell specific apoptotic effect of ADSC-CM on cancer cells, we also analyzed the activation of caspases and the levels of Bcl-2 family proteins. As we know, the change of the ratio of antiapoptotic protein versus proapoptotic proteins of Bcl-2 family proteins would activate the mitochondrial apoptotic pathway. Our data suggested that Bcl-2 expression was downregulated by ADSC-CM whereas the level of Bax expression was upregulated compared to the control group. ADSC-CM treatment also caused an increase in the levels of cleaved caspase-3 and cleaved PARP which were indicative of an induction of apoptosis (Figure 6).

### 3.6. ADSC-CM Modulated Akt Pathway within Cancer Cells

**Figure 7:**
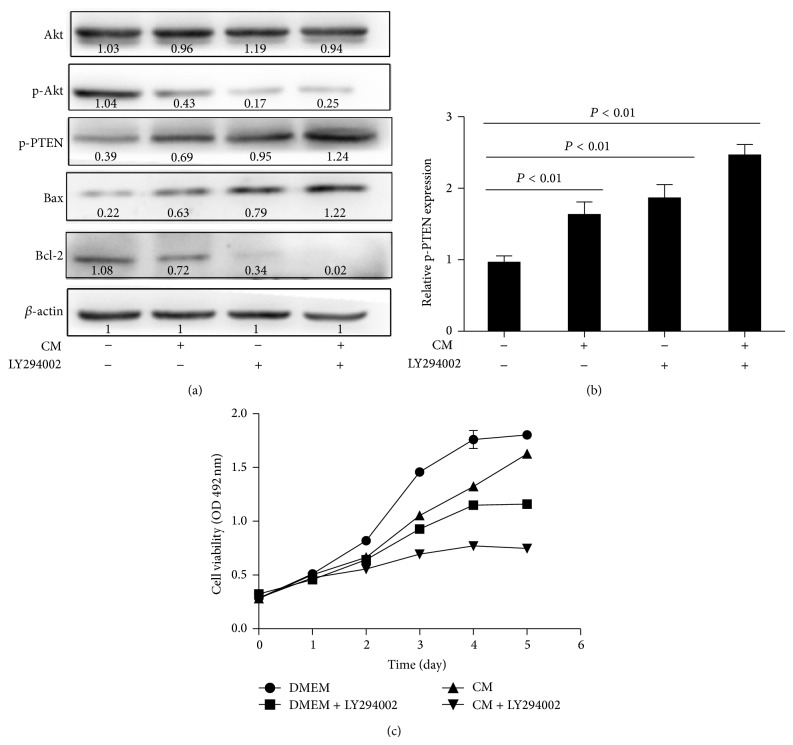
(a) T24 cells was treated with 20 *μ*M PI3 kinase inhibitor LY294002 for 60 min and then with ADSC-CM or DMEM for another 24 h. LY294002 decreased the protein levels of Bcl-2 and p-Akt and increased the protein levels of Bax and p-PTEN. Data presented in (b) are the mean ± SD of three independent experiments (^*^ *P* < 0.05). LY294002 enhances ADSC inhibition of T24 cells growth. T24 cells were treated with ADSC-CM, 20 *μ*M LY294002, or both for 24 h. Cell viability was determined by the MTS assay (c). Data shown are the mean ± SD of three independent experiments.

Recently, many studies showed PI3K-Akt can inhibit apoptosis and promote cell survival via multiple pathways [20, 21]. To determine whether ADSC-CM induced apoptosis by this pathway, we investigated the expression of p-Akt (phosphorylation of Akt) and total Akt after treatment with ADSC-CM for 48 h. Western blot analysis showed expression of p-Akt was decreased while no significant difference existed in total Akt. These results indicated that PI3K/Akt pathway played an important role in the ADSC-CM induced apoptosis in bladder cancer cells. To deeply investigate the modulation of ADSC-CM on PI3K/Akt pathway, we suppressed Akt activity with LY294002, a PI3K specific inhibitor. Cell viability was assessed with the MTS assay and protein expressions were analyzed by Western blotting. The results indicated that LY294002 enhanced ADSC-CM induced expression changing of Bax, Bcl-2, and p-PTEN (Figure 7(a)). As shown (Figure 7(b)), ADSC-CM combined with LY294002 treatment inhibited cancer cells growth more significantly than ADSC-CM alone. This implied the PTEN/PI3K/Akt signaling pathway played an important role in ADSCs' antitumor effects on bladder cancer cells.

## 4. Discussion

Recently, using ADSCs as a vehicle in tumor gene therapy had been proposed [22–25]. ADSCs' antitumor effect had been described in many studies. The mechanisms referred to the fact that ADSCs might possess several characteristics which made them be recruited to tumor and then affect cancer cells viability. The interactions between ADSCs and cancer cells, especially the secreted soluble factors, played an important role. These cytokines secreted by ADSCs (such as DKK-1, SDF-1, TGF-*β*, and RANTES), prostaglandins (such as PGE2, PGI2, and PGJ2), or interleukins (such as IL6, IL8, GSF, and IL11) were known to exert an antiproliferative or proapoptotic effect [26–30].

Our findings demonstrated the ability of ADSCs sampled from different donors to inhibit cancer cell phenotypes in two experimental systems. First, the coculture experiments reveled that ADSCs had a significant antiproliferative effect on cancer cells. Then, in order to determine whether some secreted factors were involved in this inhibition, we treated cancer cells with conditioned media of ADSCs. We found the growth of cancer cells was inhibited and the rate of apoptosis was higher in these experimental groups. Apoptosis and cell cycle arrest represented two different mechanisms involved in the induction of cell death. It was established that loss of cell cycle checkpoints was a hallmark of cancer cells, which would lead to oncogenic transformation and abnormal proliferation [31]. In our experiment, cell cycle was arrested at S phase without progressing into G2-M phase. The data suggested that ADSC-CM treatment made the cancer cells unable to accomplish normal mitosis and then undergo apoptotic death. This result was consistent with observations of our previous MTS assessment. Blocking of S phase of T24 cells cell cycle had already been reported by many articles and it may be associated with downregulation of Bcl-2 [32–35].

Finally, we characterized some of the molecular events including Bax, Bcl-2, cleaved caspase-3, and cleaved PARP which may be associated with soluble factors from ADSC-CM. The Bcl-2 protein family consisted of antiapoptotic protein (Bcl-2) and proapoptotic proteins (Bax and Bad) that regulated cytochrome c release, mitochondrial outer membrane integrity, and caspase activation leading to apoptosis. Breaking the balance of Bcl-2 family members would result in proapoptotic effects in which caspase-3 is activated and executes the apoptotic program [36]. The present study indicated that ADSC-CM treatment downregulated the antiapoptotic protein Bcl-2 while upregulating the proapoptotic protein Bax. The changes could induce cytochrome c release from mitochondria and then cytosolic cytochrome c binding to Apaf-1 and activation of caspase-3 and PARP [37]. In our research, we confirmed that ADSC-CM could activate caspase-3 and lead to PARP cleavage.

PI3K/Akt was one of the most effective antiapoptotic survival pathways and also an important cell survival factor in several types of cancer, including bladder cancer. There have been studies demonstrating that expression of Bcl-2 increased at the time when PI3K/Akt pathway was activated [38]. Meanwhile, Akt also inhibited apoptosis through mitochondrial pathways [39]. PTEN, a potent tumor suppressor which could inhibit PI3K/Akt signaling and reduced PTEN protein expression, was commonly found in bladder cancer [40–42]. So we considered that Akt might be involved in this process and ADSC-CM might inhibit PI3K/Akt signaling by upregulating p-PTEN. Consistent with our hypothesis, our data showed there was a significant inhibition in p-PTEN and phosphorylated Akt (at S473). Here we proved ADSC-CM treatment induced T24 cells apoptosis via PTEN/PI3K/Akt pathway and Bcl-2 family. Moreover, we observed cancer cells treated with a combination of ADSC-CM and LY294002 (a PI3K inhibitor) showed an increased growth inhibition compared with ADSC-CM alone. This implied that ADSC-CM could help counter resistance when used together with existing cancer therapies.

## 5. Conclusion

For the first time, we have provided the evidence to prove that ADSCs could obviously inhibit the proliferation of bladder cancer cells through apoptosis. The antiproliferative effect of ADSCs on bladder cancer cells appears to be mediated by the secretion of soluble factors which are involved in the PTEN/PI3K/Akt signaling pathway. Since ADSCs can be easily obtained as a stem cell source without ethical concerns and can be amplified quickly, ADSCs may provide a promising and practicable manner for bladder cancer therapy. However, further in vivo studies are needed to provide a more comprehensive insight into its antitumor effect.
